# In-situ collagen injection in postamputation symptomatic neuroma: A case report

**DOI:** 10.1097/MD.0000000000042107

**Published:** 2025-04-18

**Authors:** Calogero Malfitano, Giorgio Ferriero, Francesco Negrini

**Affiliations:** a Department of Biomedical Sciences for Health, University “La Statale,” Milan, Italy; b Azienda di Servizi alla Persona Istituti Milanesi Martinitt e Stelline e Pio Albergo Trivulzio, Milan, Italy; c Istituti Clinici Scientifici Maugeri, Institute of Tradate, IRCCS, Tradate, Varese, Italy; d Department of Biotechnology and Life Sciences, University of Insubria, Varese, Italy.

**Keywords:** amputation, collagen, neuroma, rehabilitation, ultrasound guided injection

## Abstract

**Rationale:**

Postamputation pain remains a significant clinical challenge, affecting a substantial proportion of individuals with limb loss. Among its etiologies, symptomatic neuromas—resulting from aberrant peripheral nerve regeneration—are a common source of residual limb pain. These lesions often lead to severe discomfort and a considerable decline in the quality of life. Although surgical treatments are available, they are often invasive and carry a risk of complications, especially in elderly or frail patients. Nonsurgical interventions, such as steroid injections, provide temporary relief but lack long-term efficacy.

**Patient concerns:**

This case study investigates the use of in situ collagen injections as a novel treatment for symptomatic neuroma in an 87-year-old patient with a longstanding transfemoral amputation. The patient had experienced similar symptoms multiple times before, marked by exacerbation and partial remission periods. However, these symptoms have become more intense and disabling over the past 3 months.

**Diagnoses:**

A thorough neurological evaluation revealed no overt motor or sensory deficits aside from pain, hypersensitivity, and tenderness above a soft tissue mass on the posteromedial side of the transfemoral stump. Elicitation of Tinel’s sign reproduced both phantom and residual limb pain. A diagnosis of symptomatic postamputation neuroma was established based on clinical criteria and further corroborated by ultrasound imaging, which allowed direct visualization of the neuroma.

**Interventions:**

The therapeutic protocol involved 2 ultrasound-guided perineuromal injections of a porcine-derived collagen medical device, administered 3 days apart. Pain intensity and sonographic characteristics of the neuroma were assessed over a 12-week follow-up period.

**Outcomes:**

Results indicated a significant reduction in pain 1 week after the second injection, with improvements lasting up to 4 weeks. Functional outcomes, including prosthetic tolerance, also showed improvement. However, the treatment’s effect diminished by the 12-week follow-up.

**Lessons:**

This case highlights the potential utility of in situ collagen injections as a safe, minimally invasive therapeutic option for symptomatic neuromas. Although the observed analgesic and functional benefits warrant further investigation, collagen injections could offer a new therapeutic approach for managing neuroma pain and serve as a promising alternative to more invasive interventions, especially in the elderly and multimorbid patients.

## 1. Introduction

Postamputation pain remains a significant clinical challenge, affecting a substantial number of amputees and impairing their quality of life.^[1]^ There are 2 main types of postamputation pain: residual limb pain (RLP) and phantom limb pain, with an estimated 95% of people with amputations experiencing 1 or both.^[2]^ These 2 categories distinguish pain based on its perceived location: phantom limb pain is pain felt in the absent part of an amputated limb, and RLP is pain experienced in the remaining part after amputation.

RLP, formerly known as “stump pain,” often has an identifiable anatomic correlate, such as wound healing abnormalities, infections, musculoskeletal issues (e.g., referred pain from the spine or joints, heterotopic ossification, myofascial pain) or, most commonly, pain caused by a poorly fitting prosthesis that creates excessive pressure points.^[3]^ Pain is typically described as sharp, burning, electrical-like, or “skin-sensitive”; it can be localized to a superficial incision, be perceived deep in the residual limb, or sometimes encompass the whole residual limb. In some cases, the RLP is related to a neuroma that develops due to an abortive attempt to repair the nerve stump after amputation. This regrowth leads to the formation of disorganized nerve fibers surrounded by dense scar tissue that is symptomless. The neuromas are estimated to be symptomatic in a percentage of patients ranging from 4% to 49%.^[4]^ They can become very painful and debilitating, hindering prosthetic use and reducing quality of life.^[5]^ Sensory dysfunctions such as hypesthesia, dysesthesia, paresthesia, hyperalgesia, allodynia, and anesthesia in the affected area of the nerve could be present.^[6]^

Due to the focal nature of neuroma pain, surgical approaches – such as traditional neuroma excision or newer reconstruction techniques based on transferring the severed nerves to a target muscle – have shown promising efficacy.^[7]^ However, these approaches are not yet part of the standard procedure, can be fraught with surgical complications, and require a recovery period before the patient can return to using the prosthetic limb.^[8]^

There is still no consensus on the gold standard nonsurgical treatment, although injections of various medications have been proposed.^[4]^ A case report of an amputee with a painful neuroma demonstrated that perineural injection with lidocaine and corticosteroid results in pain relief to a level manageable with oral opioids, but the effect was short-lasting.^[9]^ In subsequent work,^[10]^ injections of steroids into the painful neuroma improved RLP at rest and during prosthesis use in 50% of patients; however, those who experienced improvement were closer to the date of amputation and had a shorter prior duration of pain symptoms. Other nonsurgical approaches include radiofrequency ablation and cryoablation,^[11–13]^ but the effectiveness of these treatments is questionable, and there is a lack of reliable evidence.^[4]^

Since a reliable gold-standard nonsurgical treatment of postamputation symptomatic neuroma is lacking, there is still ample space for experimenting with new and promising treatments. Recent advances in tissue engineering and regenerative medicine have focused on biologically active materials to promote nerve regeneration and reduce pain. Collagen, a naturally occurring protein found in connective tissues, has shown promise as a bioactive scaffold that supports nerve healing.^[14,15]^ Injectable collagen-based medical devices are now being explored for their potential to address symptomatic neuromas. For instance, a mini-series involving 5 patients with Morton’s neuroma showed progressive amelioration in pain and function scores over a 6-month follow-up period.^[16]^ In this scenario, we hypothesize that injectable medical devices based on collagen use are a promising avenue for postamputation pain management. The rationale stems from collagen’s positive effect on reducing the inflammatory process by blocking pronociceptive neurochemicals in experimental models and providing mechanical strength to support tissue.^[14]^ To the best of our knowledge, no papers in the literature explore using collagen-based devices in this field. The following case report aims to assess the feasibility and safety of in situ collagen injection in postamputation symptomatic neuroma. It adheres to the case reporting guidelines structure and reporting guidelines.^[17]^

## 2. Case presentation

### 2.1. Patient information

**Figure 1.**
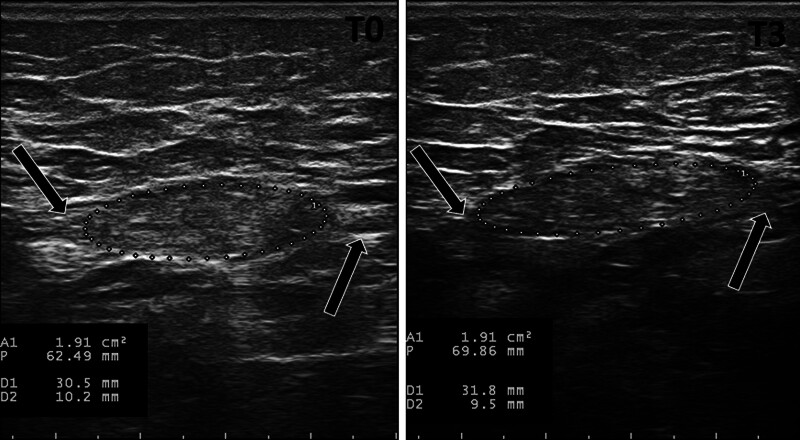
Ultrasound image of the symptomatic neuroma visualized in the longitudinal plane on the posteromedial side of the transfemoral area stump. T0: before the treatment. T3: at 3-month follow-up. Dotted ellipse: area of the postamputation neuroma. Arrows: proximal and distal intact nerve segments.

An 87-year-old man who had a traumatic left transfemoral amputation at the age of 7 was admitted to our Institute’s outpatient amputee clinic due to reduced ambulatory autonomy caused by persistent stump pain. He had been pain-free for several years, was doing well with his prosthesis, and led a very active life. In the last 5 years, he began complaining of “throbbing” pain in the residual limb, associated with dysesthesia and allodynia, rendering prosthesis use impossible for long periods and affecting his ambulation. The patient had experienced the same symptoms several times before the current admission, with exacerbation and partial remission periods. Still, during the last 3 months, they were more intense and disabling. Initially, the pain was attributed to the prosthesis fitting, and adjustments were made; however, the pain persisted. During the last years, various medications were administered, including gamma-aminobutyric acidergic and neuroleptic therapy, which did not yield positive results or were poorly tolerated. At the time of our first evaluation, he was on a regimen of amitriptyline at 10 mg per day and received nonsteroidal anti-inflammatory drugs (NSAIDs) as needed. Specifically, he received intermittently intramuscular Diclofenac at a dose of 75 mg once daily for approximately 4 days per week in the 3 months before, with limited symptomatic relief. His medical history was unremarkable, aside from hypertension and a past occurrence of ischemic heart disease. Cognitive functions were assessed as normal. A thorough neurological assessment ruled out motor and sensory deficits in the non-affected limbs. During the physical examination, a painful soft tissue mass was observed on the posteromedial side of the transfemoral stump; palpation of the mass reproduced some of the patient’s symptoms, and hypersensitivity and tenderness were noted in the stump area above the mass. Additionally, he exhibited a positive Tinel’s sign, which triggered phantom and RLP upon percussion. We also performed an ultrasound examination that revealed a predominantly hyperechogenic elliptical structure (with a maximum diameter of about 3 cm and an area of 1.9 cm²) that maintained the morphology of a typical but swollen nerve, confirming the presence of a symptomatic neuroma (Fig. 1). Increasing the probe pressure during the neuroma scan replicated Tinel’s sign. The patient’s clinical presentation and ultrasound findings were highly characteristic of a symptomatic neuroma, with no red flags indicating potential progression; therefore, we did not perform further instrumental assessments. Routine laboratory blood tests showed no abnormalities, excluding other potential causes of RLP and systemic disease. Considering the patient’s age, and poor tolerance to pharmacological therapy, we propose to treat the symptomatic postamputation neuroma with in situ collagen injection. No variations in the pharmacology therapy were applied for the study period (steady consumption). However, the patient was not forbidden to receive NSAIDs if deemed necessary. The patient has provided informed consent for the publication of the case.

### 2.2. Therapeutic approach

The treatment consisted of 2 perineuromal injections, 3 days apart, with Collagen-based Medical Devices type I porcine collagen (Guna, MD Neural, total volume 4 mL). The collagen injection was delivered using an insulin syringe and a 30-gauge needle, guided by ultrasound with a 12 to 15 MHz linear transducer.

### 2.3. Outcomes

The assessments were conducted at 5 different time points: at the time of the enrollment (TX), 3 weeks after TX, right before the initial treatment (T0), 1 week after the second injection (T1), 4 weeks (T2), and 12 weeks after the treatment (T3).

In each evaluation, the patient was asked to assess the average pain level experienced over the previous 24 hours on an 11-point numerical scale (Numerical Rating Scale [NRS] 0–10, 0= = no pain, 10 = the most intense pain imaginable). Additionally, he was requested to complete the neuropathic pain symptom inventory (NPSI) and the McGill Questionnaire-Short Form (MPQ-sf), validated in the patient’s language. The NPSI is a self-reported questionnaire designed to evaluate the presence and severity of pain symptoms typically associated with neuropathic pain during the previous 24 hours.^[18,19]^ It consists of 10 items that measure different aspects, such as spontaneous pain (burning or deep), paroxysmal pain (electric shocks), and evoked pain (allodynia or hyperalgesia). Each item is scored on an 11-point NRS (0–10). In addition, 2 items measured the number of pain attacks and their duration in a range of 0 to 5, so the total score is 0 to 110, with higher scores indicating more severe neuropathic pain symptoms. The MPQ-sf was also administered to evaluate pain during the last week.^[20]^ The MPQ-sf consists of 15 descriptors of pain, including 11 from sensory categories and 4 from affective categories; descriptors are rated on an intensity scale (1 = none, 2 = mild, 3 = moderate, and 4 = severe, for a total score ranging from 15 to 60). NSAID consumption and functionality were also evaluated clinically at each time point. We also monitored the neuroma ultrasound features (longitudinal diameters and area) to detect treatment-related changes.

### 2.4. Results

A change greater than the minimal clinically important difference (MCID) was considered clinically significant. As described in a previous work,^[21]^ the threshold for MCID was a reduction of at least 30% for NRS,^[22]^ 6 points for the 15 to 60 MPQ-sf,^[20]^ and 20% of the baseline values for NPSI.^[21]^

No adverse events were observed. The pain NRS remained constant between TX and T0 (stable state). A clinically significant improvement (i.e., a variation greater than MCID) was observed 1 week after the second injection (T1 vs T0) and was sustained until the first follow-up (T2). Similarly, the MCID was statistically significant in T1 versus T0 and T2 versus T1 for both the NPSI and MPQ-sf scores. There was no significant change in neuroma size across all time points (Fig. 1). With less discomfort, the patient began using the prosthesis again and resumed walking as before the onset of pain years ago. After the treatment, the administration of 75 mg intramuscular diclofenac was reduced from about 4 doses to 1 dose per week. The treatment’s effect completely regressed after 3 months (T3) (Table 1).

**Table 1:** Clinical results at the 5 time points.

	TX	T0	T1	T2	T3
NRS	8	8	2*	4†	8
MPQ-SF	22	21	15*	16	23
NPSI	57	53	42*	43†	58

MPQ-SF = McGill pain questionnaire short-form, NPSI = neuropathic pain symptom inventory, NRS: Numerical Rating Scale, TX = at the time of the enrollment, T0 = before the treatment, T1 = 1 week after the treatment, T2 = 4 weeks, T3 = 12 weeks after the treatment.

* Score change exceeding the minimal clinically important difference (MCID) between T0 and T1.

† Score change exceeding the MCID between T0 and T2. In all 3 questionnaires, a higher score indicates a worse condition.

## 3. Discussion

Postamputation pain significantly affects amputees’ quality of life, and symptomatic neuromas are often challenging to manage with traditional treatments.^[4,7]^ Despite various surgical and nonsurgical interventions, long-lasting relief remains elusive, particularly for older or frail patients who may not be suitable candidates for more invasive procedures. The case study illustrates the potential of collagen-based injectable medical devices in managing symptomatic neuromas in a patient with a long-standing history of transfemoral amputation. The patient experienced significant reductions in pain scores, with improvements observed 1 week after the second injection (T1), which were sustained for 4weeks (T2) and diminished after 3 months (T3). During this period, the patient also experienced improved overall function, including increased prosthetic tolerance and decreased NSAID medications. To the best of our knowledge, this is the first study that describes the use of injectable collagen for the treatment of symptomatic postamputation neuroma. The only work in the literature that we can refer to for analogies is the study by Giarda et al, a clinical series on Morton’s neuroma.^[16]^ However, these 2 conditions are not entirely comparable, and some differences must be taken into account when evaluating the generalizability of their findings on Morton’s neuroma to postamputation neuroma. For example, in our clinical case, the neuroma measured a maximum diameter of about 3 cm, exceeding the typical size range of Morton’s neuroma (0.5–1 cm). Consequently, a higher dose of collagen was administered per injection (4 mL vs 2 mL) and in total (8 mL vs 6 mL). Additionally, injections were spaced 3 days apart instead of 1 week, reducing the number of administrations (2 vs 3), in accordance with the findings of Randelli et al,^[14]^ who reported peak fibroblast activation within 48 to 72 hours after collagen injection. However, unlike Giarda, where variables improved progressively, with benefits lasting until the last 6-month follow-up, in our case, the effect was immediate but regressed at the 3-month follow-up. The difference in schedule likely influences the duration of the effect, and the treatment requires repetition of the injection for sustained benefits. This may suggest that the optimal timing for collagen injections lies between 72 hours and 1 week, with a shorter period able to increase the velocity of improvement in pain and functionality. On the other hand, administering 3 injections may be more beneficial for a longer period than just 2 injections.

Furthermore, they found a reduction in pain at rest and during weight-bearing activities, thus hypothesizing a mechanism providing physical support to tissues. On this basis, the expected effect on the symptomatic amputee neuroma should be the reduction in allodynia due to the decrease in local pressure effects. Surprisingly, analysis of the NPSI revealed an unexpected improvement in paresthetic pain without notable changes in allodynia. This finding adds an interesting dimension to the discussion, suggesting that collagen has more than a simple mechanical effect. Based on existing literature,^[14]^ collagen’s bioactive properties, specifically its ability to modulate the action of peripheral pro-inflammatory immunological factors and other neurochemical substances, are plausible. Indeed, ultrasound parameters of the neuroma (such as volume and area) remained stable throughout the treatment period. This observation reinforces that collagen’s therapeutic benefit may stem more from its biological activity than any direct structural modification of the neuroma.

Another crucial observation pertains to the delayed onset of symptoms related to neuroma, which occurred in this patient over 80 years after the amputation. However, it is well-documented that neuromas can remain asymptomatic for long periods and may suddenly become symptomatic later in life due to various factors. These involve alterations in stump anatomy, the use of prosthetics, hypersensitivity of the peripheral nerve, and the mechanisms of supraspinal and cortical sensitization, as supported by pertinent literature.^[2,23]^ This underscores the complex pathophysiology of neuroma pain and highlights the necessity for longitudinal monitoring and a comprehensive approach to managing the health of residual limbs in amputees, even in cases of prolonged prosthetic use tolerance.

This case report has several limitations. Most notably, we did not directly measure the functional limitations resulting from neuroma pain; we only considered the potential for wearing the prosthesis and restoring walking as functional outcomes. Additionally, the follow-up was limited to 3 months because pain levels increased at T3 to intensities similar to those before treatment, leading us to conclude that an extended follow-up period would not be necessary. As discussed above, it is clear that various protocols (such as increased injections and higher volume/dose of collagen) are needed to evaluate the durability of the treatment effects, and longer follow-up in future studies is essential.

## 4. Conclusions

Although this is a single case, and the results must be confirmed in the future by more methodologically robust studies, our study demonstrates a clinically significant reduction in postamputation neuroma pain. The treatment seems extremely promising since it was effective on RLP while being minimally invasive, well-tolerated by the patient, and with minimal risk of adverse effects. Considering there is still no consensus on gold standard nonsurgical treatment of RLP due to neuroma, collagen injections could potentially be regarded as first-line therapy, especially in elderly and multimorbid patients.

## Author contributions

**Conceptualization:** Calogero Malfitano.

**Formal analysis:** Calogero Malfitano.

**Investigation:** Calogero Malfitano, Francesco Negrini.

**Methodology:** Francesco Negrini.

**Supervision:** Calogero Malfitano, Giorgio Ferriero.

**Writing – original draft:** Calogero Malfitano, Francesco Negrini.

**Writing – review & editing:** Calogero Malfitano, Giorgio Ferriero, Francesco Negrini.
